# Blockchain technology in migrant and refugee health: A scoping review

**DOI:** 10.7189/jogh.12.04047

**Published:** 2022-05-14

**Authors:** Ana Corte-Real, Tiago Nunes, Paulo Rupino da Cunha

**Affiliations:** 1 Laboratory of Forensic Dentistry, Faculty of Medicine, University of Coimbra, Coimbra, Portugal; 2 CISUC, Department of Informatics Engineering, University of Coimbra, Coimbra, Portugal

## Abstract

**Background:**

The increase of forcibly displaced people worldwide is a challenge for health systems and their ability to provide access and equity in Health as a universal right. In the case of migrants and refugees, their journey exacerbates this challenge, as they go through diverse countries, camps, and humanitarian teams. Hence, the collection and analysis of health data are essential in providing quality care. The scientific community has been studying health digital technologies to answer health data consolidation, transparency, and global surveillance efficiency issues. Observing some empirical experiments with Blockchain in migrants and refugee health, we assessed the state-of-the-art by conducting a literature review.

**Methods:**

Blockchain applications are still emerging, which means that peer-reviewed literature may still be scarce in life science databases. Therefore, to gather the most appropriate available evidence, we used a diverse and balanced set of databases that compile articles and journals from different fields. We used a multi-step scoping review to refine search keywords and analyse the literature. We included studies between 2008 and 2021 that reported value, utility, or use cases of Blockchain in support of migrant and refugee health.

**Results:**

We identified a total of 69 articles, with 22 retained for full-text analysis and 8 of those being relevant. We employed Rayyan application to manage and evaluate the references by two researchers working independently. We identified two main uses of Blockchain technology to support migrant and refugee health: mitigate the lack of personal identification and make health records available. Blockchain also promotes data reliability in humanitarian aid, academic certificates, legal contracts, and financial transactions.

**Conclusions:**

The availability of reliable information about individuals facilitates universal health coverage, improves cooperation between diaspora-related countries, and supports global health efficiency in line with the third goal of the Sustainable Development Goals 2030 agenda. Given its characteristics of decentralization, resilience, transparency, and auditability, Blockchain remains a promising avenue for future research in migrant and refugee health.

Since the World Health Organization (WHO) constitution in 1946, the right to health has been increasingly recognized and has gained preponderance in the international agenda [1]. On the other hand, according to a 2010 report from the International Organization for Migration (IOM), international migrants worldwide may be as many as 405 million by 2050 [2]. This trend stems from a-significant increase in people's mobility, fostered by globalization, low transportation costs, economic pressures, demographic trends, environmental degradation, violence, and human rights abuse [2].

Current approaches to managing the health of migrants struggle to keep up with the growing challenges associated with the complexity, volume, speed, diversity, and disparity of current migration flows. As a result, migrants’ fundamental right to health is at risk. The 2010 WHO report calls for the delivery of health services to migrants in a culturally and linguistically appropriate manner and for capacity building of the health and relevant non-health workforces to address key issues such as vaccination [3]. However, collecting, compiling, maintaining, assuring quality, analysing health data records and statistics on migrants, refugees, asylum seekers, internally displaced persons, and stateless people remain challenging [3,4]. The same can be said about the interpretation of global indicators [5]. The scarcity of reliable data on migration and health, particularly about undocumented people, has also been noted by the WHO [6]. It can be a significant reason for the lack of understanding of migrants' and refugees' health needs [7].

Achieving universal health coverage, including access to quality essential health care services, is a target in the third Sustainable Development Goal (SDG) of the 2030 Agenda [8]. However, according to legal law, identity data and authentication are essential to recognize migrants' existence and for them to exercise rights and access services, namely health care. The magnitude and severity of the absence of data on these vulnerable populations hinders access to health care services, influencing the achievement of the SDGs [4]. Personal identification and inclusion are also a cornerstone of patient-centred medicine.

So far, international guidelines for data collection and analysis have been implemented as an appropriate procedure for data comparison and to support quality assessment in health systems [9]. This gradual implementation, progressing from national to international scopes, in a global context, will improve health systems' capability to assess the impact of the health policies among different social groups, namely migrants. Nevertheless, the Global Consultation on Migrant Health in Madrid, in March 2010, called for the strengthening of information systems to collect and disseminate migrant health data, disaggregated by categories, to effectively evaluate health indicators while safeguarding ethical issues [10]. Traditionally, such databases are centralized and controlled by a trusted third party who owns the data. However, this approach raises several issues, such as difficult access to the data by stakeholders (eg, the individual or other health organizations) and low resilience to destruction or tampering, namely in case of conflicts or hacking. In 2021, in the M8 Alliance Expert Meetings about Digital Solutions for Migrant and Refugee Health [11], we have discussed the potential of Blockchain technology to address these concerns. Blockchain, originally introduced in the context of Bitcoin to avoid the double-spending of digital money, has been applied to multiple other areas where trust is a key concern [12].

Blockchains are a type of distributed ledger technology (DLT) that allows data to be securely recorded and distributed across a network of peers in different institutions and locations. Adding data to a Blockchain requires a consensus. A transaction is generated and broadcasted for agreement among the peers. After being validated, the transaction is added to a pool of unconfirmed transactions. A set of unconfirmed transactions is collected into a new block. A consensus algorithm is executed, the new block is added to the Blockchain, and the peers are updated. As a result, the records become untamperable, and information can only be added, not removed, nor changed [13]. The commonly used Proof-of-Work consensus algorithm requires the peers to find the solution to a computationally-intensive puzzle and get rewarded with an amount of the associated token, or cryptocurrency, and transaction fees [14]. More recent consensus mechanisms, such as Proof-of-Stake (PoS), do not involve computationally intensive operations and thus can reduce power consumption by more than 99%. In these cases, processing power is replaced by deposited and locked capital or tokens [14]. Proof-of-Stake has various advantages, such as better energy efficiency and reduced hardware requirements [15]. The decentralization of ledgers helps resolve various issues related to centralized databases, like the need for a trusted third party, which is also a single point of failure.

More modern Blockchains also support the storage and enforcement of smart contracts, pieces of computer code that execute automatically when previously agreed-upon terms are fulfilled. This feature can be used to automate any process, enhancing transparency, [13] and further promoting trust among participants of a transaction in the absence of intermediaries or trusted third parties [16].

Given its development prospects and characteristics of decentralization, resilience, transparency, and auditability, Blockchain holds potential for many areas of development, including Fintech, Internet of Things, cloud, and health care [17-19]. Some uses of Blockchain in health care, highlighted in the literature, are managing hospital overheads or even creating and managing patient medical records, shared and linked to multiple parties, efficiently and transparently [20].

Blockchain technology as a digital solution to support migrant and refugee health is a recent subject in the scientific community. It was first mentioned in the extant literature in 2018 in an article that examined Blockchain applications in migration and diaspora-related initiatives in the context of new digital technologies in globalization and transnational spaces [21]. Since then, more articles have been published, and the array of use cases has been growing.

Having observed some empirical experiments with the Blockchain in support of migrant and refugee health, we decided to perform a literature review to find out the state-of-the-art in the academic literature, which led us to the following research question (RQ):

**RQ:** What is the state-of-the-art in the use of Blockchain in migrant and refugee health?

To answer this RQ, we decided to perform a scoping review, as described in the next section.

## METHODS

To provide an overview of the existing literature on the topic of the RQ, we performed a scoping review following the Arksey & O'Malley [22] framework for scoping studies. To identify relevant scientific literature rigorously and transparently and ensure consistency in decision-making, we also used guidelines for systematic reviews described by Webster & Watson [23] and Kitchenham [24].

We used MEDLINE (accessed through PubMed) and Cochrane Library to cover biomedical and life sciences papers, the ACM Digital Library, IEEE Xplore, and AISeL to account for papers focused on engineering and technology, and ScienceDirect and Wiley Online Library due to their broad coverage.

The inclusion criteria were conference and journal articles written in English, Portuguese, Spanish, and French. We considered papers published after 2008, the year of Satoshi Nakamoto’s paper on Bitcoin, the first successful implementation of Blockchain technology [25]. The search was carried out in June 2021.

We started (Step 1) by searching different combinations of the keywords “Blockchain”, “Migrants”, “Refugees”, and “Health”, derived from the scope of our research. We present the resulting search expressions in **Table 1**. We used minor variations to account for the different user interfaces of the various databases.

**Table 1:** Search Expression for Step 1

Step 1 Search Expression
Blockchain AND migrants AND Health
Blockchain AND refugees AND Health
Blockchain AND (migrants OR refugees) AND Health

**Figure 1:**
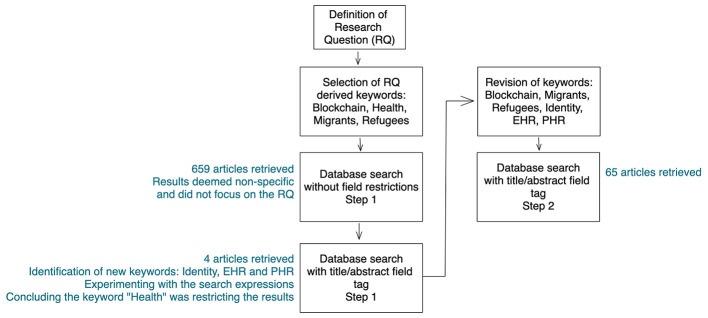
Flowchart of the study search process.

Searching without strict limitations or restrictions in any field retrieved a total of 659 articles. However, by reading the titles and abstracts, we noticed the results were non-specific, and most did not match the subject of our study. For example, when the keywords (such as Blockchain) are mentioned in passing among many others such as “artificial intelligence,” “Internet-of-Things,” and other contemporary technologies. As noted by Arksey & O'Malley [22], the methodology in a scoping study is not a linear process, and each stage should be engaged reflexively, and, if needed, some steps may be repeated. Therefore, we added the field tag [Title/Abstract] for added focus on the research question. As a result, we obtained only four articles reporting the use of Blockchain to support the health of migrants and refugees, namely proposing the use of this technology to address the issues of lack of personal identification and unavailability of health records. These results led us to recognize “Identity”, “Electronic Health Records” (EHR), and “Personal Health Records” (PHR) as relevant keywords to be used in our search strategy. By experimenting with the search expression, we also noticed that the inclusion of the keyword “health” was restricting the results since 13 articles mentioned the use of Blockchain technology in the context of migrants and refugees, but only two mentioned health. Considering these results, we decided to add a second step (Step 2) to our literature review. We removed the keyword “Health” and added “Identity”, “EHR”, and “PHR”, leading to the different combinations in **Table 2**. We maintained the same set of databases and inclusion criteria. **Figure 1** illustrates the search process with the number of articles retrieved and the keywords used at each step.

**Table 2:** Search Expressions for Step 2

Step 2 Search Expression	
Blockchain AND Migrants	Blockchain AND Refugees
Blockchain AND (Migrants OR Refugees)	Blockchain AND Refugees AND Identity
Blockchain AND Migrants AND Identity	Blockchain AND (Migrants OR Refugees) AND Identity
Blockchain AND Migrants AND HER	Blockchain AND Refugees AND EHR
Blockchain AND (Migrants OR Refugees) AND EHR	Blockchain AND (Refugees OR Migrants) AND PHR
Blockchain AND migrants AND PHR	Blockchain AND (Migrants OR Refugees) AND (EHR OR PHR)

After reviewing the results, we also searched the grey literature for supporting data, gaps, and possible “new frontiers” yet to be discussed in the scientific literature. We found news articles, highlights, and reports on websites of international organizations focused on health issues and organizations with on-going data-recording and identification initiatives including Blockchain technology.

## RESULTS

We identified a total of 69 articles in both steps of our search: four on Step 1 and sixty-five on Step 2. After removing the duplicates on both steps and double-checking after merging the two sets of results, 22 articles remained for analysis.

We employed Rayyan QCRI [26] to manage the references. Using this tool, two reviewers independently evaluated and classified each article, based on the title and abstract, into one of three categories: 0 – exclude; 1 – maybe; 2 – include. We resolved eligibility disagreements among the reviewers in Zoom meetings by presenting and discussing the rationales for the assigned classifications. This kind of researcher triangulation is essential to increase validity and decrease biases [27,28].

**Figure 2:**
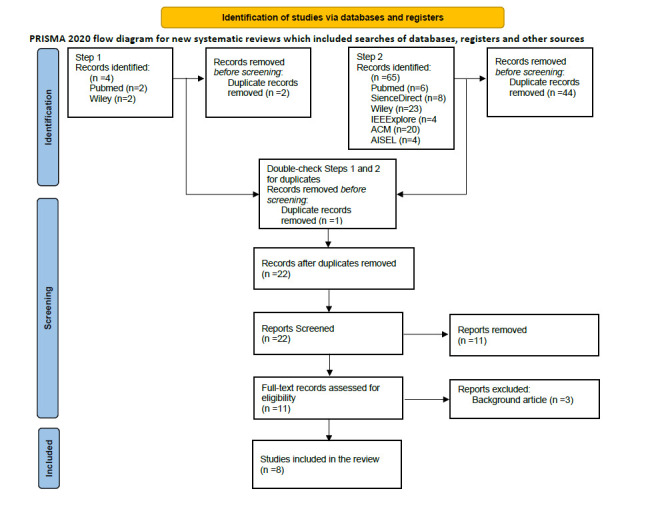
Flow diagram of the study selection.

Title and abstract screening excluded 11 articles, and after full-text assessment, we excluded an additional three articles. We deemed 14 articles out of scope, and eight remained for further analysis and data extraction. **Figure 2** describes our literature identification process, oriented by the PRISMA 2020 flow diagram for systematic reviews [29].

The studies that satisfied the inclusion criteria were processed for data extraction and summarized in **Table 3**.

**Table 3:** Literature data of the included studies (n = 8)

Author (Year)	Type of study	Scope/Issues	Blockchain application/implications for vulnerable populations
**Rodima-Taylor (2018) [** 15 **]**	Not explicit, but authors state “In this article, we explore the implications of digital ledger technologies for global networks of value transfer and verification.”	Lack of identity Financial transactions	Estonian e-residency programme, “provide a government-authenticated digital identity to foreigners”, “Offering a transnational digital identity to citizens of any part of the globe, it allows anyone outside Estonian borders to engage in commercial activities with public and private sectors, using a ‘platform built on inclusion, legitimacy and transparency’.” “provide secure digital identity for KYC compliance and a digital fiat for currency conversion”, “reduce the costs of cross-border fund settlement in remittance transfers”, “replace corresponding bank chains (…), significantly lowering the costs and duration of remittance transactions.”
**Blue (2019) [** 16 **]**	Not explicit, but authors state “This paper outlines the design and initial development of a solution that could potentially allow refugees who are resettling to build a digital identity document over time as they settle in a new land, without the need for multiple sources of traditional identity documentation.”	Lack of identity	“design of an algorithm that aims to authenticate or refute identities based on information gained from digital footprints.”, “construct a secure and intelligent digital identification document.”, “allow individuals to assert their identity without requirement for traditional paper documents or electronic records.”
**Abraham (2019) [** 17 **]**	Not explicit, but authors state “This paper follows four activist projects (...) and the effort to build a database for undocumented Rohingyas using blockchain”	Lack of identity	“give undocumented Rohingya a digital identity through the creation of a virtual database”, “build (..) a decentralized and encrypted means for people to engage in online collective activities.”, “enable each registered person to be identified uniquely, giving them a digital identity that is secure and independent of state controls.”
**Wiatt (2019)[** 18 **]**	Blind peer review	Lack of identity. Academic records	“*store, validate, and access* [identity and academic] *degrees and certificates from remote devices*.”
**Khurshid & Gadnis (2019) [** 19 **]**	Policy proposal	Lack of identity Health records	“create profiles for persons experiencing homelessness, using biometric features.”, “secured, immutable identity created for every individual in the program.” “allow persons to access their health data and details”, “record all their transactions with health providers and emergency medical services through the same ledger.”, “allows individuals to have direct access to all transactions that are recorded on the platform through any interaction in the system while also allowing each agency to access that record with permission of individuals.
**Seyedsayamdost (2020) [** 20 **]**	Not explicit, but the authors state “This paper examines three projects that have piloted DLT use cases, (…) explores the use of blockchain technology with a view to understanding its implications for governance”	Humanitarian aid, Lack of identity Humanitarian aid, Financial transactions	ConsenSys- Moldova, “create digital identities and expand access to verifiable identification while improving security over personal data (…) that relies on biometric identifiers, including fingerprints and iris scans, which can protect minors from trafficking.” WFP’s Building Blocks, “treamline delivery of food and cash, particularly in crisis situations where speed is of utmost importance and the financial infrastructure has been disrupted and is ineffective in the delivery of life-saving resources”, “facilitate financial inclusion for billions of people by not only enabling the creation of a digital identity but also allowing people to directly transact with others.” Moeda “offers impact investors the opportunity to make capital available to those in need, while being able to expect a return on their investment, and a reasonable degree of liquidity.”
**Christ (2021) [** 21 **]**	Not explicit, but the authors state “The purpose of this paper is to offer a conceptual discussion as to how a relatively new technology, the blockchain, might be used to reduce vulnerability and risk in migrant worker populations.”	Lack of identity, Migrant labour exploitation	“provide [migrant] workers with a clear record of their identity which can then be linked back to their visa and contract of employment. Such evidence will provide protection against confiscation of identity documents which can be used as a form of blackmail to prevent workers from leaving unacceptable working arrangements and lead to forced labour”, “record and provide an auditable trail of transactions associated with labour recruitment, such as employment contracts, migration details, passports and visa arrangements”, “provides a way to ensure the initial contract terms are permanently, immutably recorded for anyone to see.”
**Shuaib (2021) [** 22 **]**	Not explicit, but authors state “This paper reviews the aspects of SSI application during the pandemic situation like COVID-19.”	Lack of identity, Access to health, Health records, Covid-19 assistance	“creation of a blockchain-based self-sovereign identity with the potential of providing medical information could significantly affect the migrants lacking on lawful documents”, “provide access to undocumented people’s fundamental rights, which should become standard and even more critical in regularly responding to crises”, “self-sovereign identity-based immunity credential to offer the tracing and tracking of COVID-19 test-takers”, “digital credentials will provide the patients with verifying their health conditions and immunity against various diseases”, “fight and control the outbreaks of COVID-19”

The type of study is as reported by the authors or inferred from the study details if not explicit. The articles address the issues: lack of identity, humanitarian aid, financial transactions, access to health, academic and health records, Covid-19 assistance, and migrant labour exploitation. We organized the issues in columns and presented them individually if addressed separately by the authors’ or grouped multiple issues in the same column if addressed as interrelated factors. We identified the Blockchain applications and their implications for vulnerable populations. Four articles introduced new concepts and proposals for Blockchain applications, while others explored running use-cases and their implications on the issues faced by migrants and refugees.

## DISCUSSION

Blockchain offers new possibilities for enterprise improvement and public sector management and communication as a disruptive technology. For example, centralized data logging systems as we know them today can move to a distributed system while ensuring no alteration of data and maintaining privacy [20].

Although the available scientific literature on the use of Blockchain in the health care sector has been summarized in previous articles, migrant and refugee health had not been covered yet.

This paper reflects the state-of-the-art of Blockchain applications in support of migrant and refugee health. We selected a scoping review with a broader research question, maintaining a comprehensive approach and identifying relevant literature regardless of study design to fulfil the absence of articles mapping the research activity on this topic.

In the scientific literature, we found two main uses of Blockchain technology in support of migrant and refugee health: addressing the lack of personal identification [21,30-36] and the unavailability of health records [30,36]. Additionally, Blockchain is also used to promote data reliability in (migrant) academic certificates [18] and legal contracts [31].

The creation of a digital identity stands out as a key Blockchain use in the context of migrant and refugee health, as is reported in every article included in our review [21,30-36]. The lack of a legal ID is a barrier to receiving health care, even when the migrants and refugees are entitled to it. The security and integrity of the information stored in a Blockchain, in this case, identity information, enables migrants and refugees to assert their identity and personal information [21,32,33]. Khurshid & Gadnis proposed the use of Blockchain to create a digital identity for homeless persons, accessible by health professionals in different cities and countries [36]. Although the article refers to the homeless, they are characterized by the authors as a vulnerable population facing identical challenges to those of migrants and refugees [36]. Shuaib et al. reviewed the applications of Blockchain for COVID-19 assistance. They argued that using this technology to create a self-sovereign Blockchain-based identity could be very useful to counter or solve the issues of anonymity, accuracy, and personal information preservation and control in a pandemic context, ensuring the fundamental rights of undocumented people [30].

Health data availability can also be improved using Blockchain to store electronic health records [30,36]. Ineffective data sharing and poor coordination lead to the duplication of clinical outcomes [7], which in turn causes global health costs to rise [36]. The higher availability of health data, enabled by the inherent distribution of data in a Blockchain, can help mitigate this problem [36].

**Figure 3:**
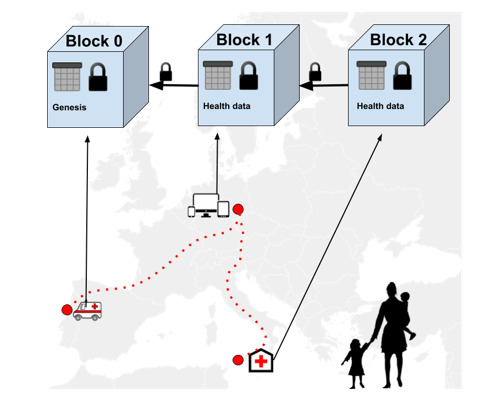
Updating health records during the migration flow.

Limited access to information and support services, namely during the pandemic, has intensified the risk of illness in displaced populations, ending in chaotic global health and socioeconomic implications. Using Blockchain, medical data are uploaded, agreed upon, and automatically distributed to all the stakeholders of a decentralized peer-to-peer network (eg, health providers, institutions, NGOs), allowing remote access to health data when required. Furthermore, the integration of smart contracts to program medical data sharing between the participants prevents communication issues and empowers patients in patient-centred health care in a cost-effective, secure, and auditable manner [37]. Besides being untamperable and therefore more reliable, records stored on a Blockchain are decentralized and under the control of the individuals, who can share them with the health care workers assisting them, as illustrated in **Figure 3** [30,36]. Migrants and refugees are thus provided with resilient and available electronic health records, populated with data resulting from interactions with health providers and institutions [30,36], such as previous diagnostics, exams, therapeutics, and even vaccination or immunity certificates.

Digital solutions for data recording should include low and medium-income countries like Bangladesh, Djibouti, Ethiopia, Iraq, Ethiopia, and Uganda [4]. The global access and recording of health data in transnational spaces improves cooperation between diaspora-related countries and overcomes local limitations of National health systems [38].

Despite the challenges in good practices guidelines and health policies between countries, global health efficiency should be ensured and continuously supported by equity health care and good patient experience. This is achieved by health quality-improvement strategies discussed by global referenced institutions in line with the third goal of the SDGs 2030 agenda [4,8]. At a Health global level, data management can be in accordance with health indicators for good practices and new health strategies implementation. A large-scale approach for severe (eg, oncological pathology) and heterogeneous illnesses (eg, COVID-19) is a health issue priority related to accurate diagnosis and treatment performance [38].

Infectious diseases, namely the rapid spread of the recent COVID-19 pandemic, have shown the relevance of validated data sharing, electronic health record management systems, and their impact on global health. However, data protection regulations remain a challenge in terms of data sharing [39]. For example, European Union's General Data Protection Regulation (GDPR) requires care in handling personal data, namely because users are entitled to withdraw consent for processing and have the right to be forgotten [40]. Similarly, California’s Consumer Privacy Act, a landmark law applied since 2018 in the business context, includes the right to delete and opt-out personal information [41]. Since information recorded on a Blockchain cannot be removed or changed, using this technology in electronic health records is challenging. There are, however, technical and legal solutions to pursue.

Compliance with privacy regulations was studied within a customized architecture design [42]. Rieger et al. presented a three-level architecture of Germany’s Federal Office for Migration and Refugees Blockchain Solution as an example of compliance with GDPR, pseudonymizing the data on the Blockchain [42]. It involves separating the digital Identity from the digital Blockchain transactions [42]. This type of hybrid solutions, where only a minimum amount of data are stored on the Blockchain, can also reduce costs and help mitigate concerns about the operation of a Blockchain, namely the supposedly inherent energy consumption and environmental impact [14].

One concern raised during our presentation in the M8 Alliance Expert Meetings about Digital Solutions for Migrant and Refugee Health [11], that is not mentioned in the literature, is the implementation of Blockchain in low-resource settings, such as refugee camps. Some existing Blockchains have high operation costs, mainly due to the need for expensive hardware and its considerable energy consumption. However, this reality is changing. For example, as part of a set of ongoing upgrades, Ethereum, one of the more popular Blockchain networks, is moving from a Proof-of-Work to a more energy-conscious Proof-of-Stake consensus algorithm. This change is part of a plan to make the project more scalable, secure, and sustainable, encouraging its adoption [15]. Ethereum has been used in humanitarian aid initiatives, namely the World Food Program’s Building Blocks [43] and ConsenSys & Moldova’s Blockchain Human Trafficking Project [44]. In the World Food Program’s Building Blocks, Blockchain and smart contracts are used to create a digital identity and deliver food and financial aid to people in crises, improving transparency, cost-efficiency, and security [32]. In ConsenSys and Moldova’s project, Blockchain is also used to create digital identities and expand access to verifiable identification that can rely on biometric identifiers, including fingerprints and iris scans, which can protect minors from trafficking while improving security over personal data [32].

Blockchain can also be used to support migrants and refugees besides health care services [7]. It can be used to store and validate academic degrees and certificates [35], which will facilitate job integration at the destination. It can be used to promote financial inclusion [21,32] thanks to new services for the traditionally unbanked. It can speed up and reduce the costs remittances in developing countries [21] and help engage in online collective [34] and commercial activities [21]. Additionally, Blockchain-based digital identities can be linked back to visas and labour contracts, preventing documentation retention and fraud, protecting migrants and refugees from ill-intentioned employers [31]. Blockchain technology can enable global companies to verify their labour supply chains [31]. Employment contract terms are permanently, immutably recorded and accessible, thereby enhancing accountability and facilitating auditability [31,45].

This improved supply chain has the potential to turn what was once considered that cheap labour was preferable at any price into a condition on human rights are protected and embedded within all labour services [31].

## CONCLUSIONS

Blockchain presents itself as a promising technology with features that can help overcome migrant and refugee health gaps and accelerate progress toward Universal Health Coverage. Although Blockchain is used in various contexts and sectors that influence the health of migrants and refugees, there are two areas where its importance is highlighted: addressing the lack of personal identification and the unavailability of health records.

Energy consumption concerns and compliance with data protection regulations are viewed as challenges to implementing Blockchain but can be potentially overcome with adjusted system designs and consensus mechanisms.

Presently, support for the use of Blockchain in health care is mainly empirical, including ad hoc implementations by companies and other organizations. The scientific literature predominantly comprises articles without a defined typology, reviewing and commenting on existing ad hoc implementations. More original research articles are needed that study other potential areas of use and allow for a more rigorous assessment of the impact of Blockchain on migrant and refugee health. We conclude that conducting a full systematic review is not yet feasible.

However, Blockchain is a promising pathway for future research in migrants' and refugees' health.
